# Reporting performance of prognostic models in cancer: a review

**DOI:** 10.1186/1741-7015-8-21

**Published:** 2010-03-30

**Authors:** Susan Mallett, Patrick Royston, Rachel Waters, Susan Dutton, Douglas G Altman

**Affiliations:** 1 Centre for Statistics in Medicine, Wolfson College Annexe, University of Oxford, Linton Road, Oxford, OX2 6UD, UK; 2 MRC Clinical Trials Unit, 222 Euston Road, London NW1 2DA, UK

## Abstract

**Background:**

Appropriate choice and use of prognostic models in clinical practice require the use of good methods for both model development, and for developing prognostic indices and risk groups from the models. In order to assess reliability and generalizability for use, models need to have been validated and measures of model performance reported. We reviewed published articles to assess the methods and reporting used to develop and evaluate performance of prognostic indices and risk groups from prognostic models.

**Methods:**

We developed a systematic search string and identified articles from PubMed. Forty-seven articles were included that satisfied the following inclusion criteria: published in 2005; aiming to predict patient outcome; presenting new prognostic models in cancer with outcome time to an event and including a combination of at least two separate variables; and analysing data using multivariable analysis suitable for time to event data.

**Results:**

In 47 studies, Cox models were used in 94% (44), but the coefficients or hazard ratios for the variables in the final model were reported in only 72% (34). The reproducibility of the derived model was assessed in only 11% (5) of the articles. A prognostic index was developed from the model in 81% (38) of the articles, but researchers derived the prognostic index from the final prognostic model in only 34% (13) of the studies; different coefficients or variables from those in the final model were used in 50% (19) of models and the methods used were unclear in 16% (6) of the articles. Methods used to derive prognostic groups were also poor, with researchers not reporting the methods used in 39% (14 of 36) of the studies and data derived methods likely to bias estimates of differences between risk groups being used in 28% (10) of the studies. Validation of their models was reported in only 34% (16) of the studies. In 15 studies validation used data from the same population and in five studies from a different population. Including reports of validation with external data from publications up to four years following model development, external validation was attempted for only 21% (10) of models. Insufficient information was provided on the performance of models in terms of discrimination and calibration.

**Conclusions:**

Many published prognostic models have been developed using poor methods and many with poor reporting, both of which compromise the reliability and clinical relevance of models, prognostic indices and risk groups derived from them.

## Background

Prognosis is central to medicine, often being used to direct diagnostic pathways and to inform patient treatment [1,2]. Most clinicians use patient and disease characteristics to predict patient outcome. For accurate outcome prediction, multiple risk factors need to be considered jointly because single factors have insufficient predictive value to distinguish patients who are likely to do well from those likely to do poorly. Prognostic models allow multiple risk factors to be used systematically, reproducibly and using evidence based methods [2-5]. Prognostic models are also referred to as clinical prediction models, clinical prediction rules, risk scores and nomograms. Although a large number of prognostic models are published, very few models are used in clinical practice [3].

To understand whether a particular prognostic model or prognostic index provides a useful tool to inform patient treatment, the accuracy of the model predictions need to be reported, both in terms of how well the model separates individuals who develop the outcome from those that do not (discrimination) and how close the predicted risks are to the actual observed risks (calibration). Currently several measures of model performance are used, although there is no consensus on which are the most clinically useful given the range of different clinical decisions directed from prognostic models [6].

This article examines how prognostic models are used to develop clinical predictions about cancer patients, and the measures of model performance that are used.

We assessed, by a systematic review, the methods used in 47 articles on prognostic models where the specific research aim was to develop a new prognostic model as a combination of at least two separate variables to predict patient outcome. We focussed on the reporting and use of methods used to develop prognostic indices and risk groups from the models and measures used to determine how well the model predicted prognosis. We have set our findings in the context of the methodological literature that has studied the impact of using different methods of model predictions.

Development of good prognostic models needs researchers to provide reliable information for patient treatment decisions, including measures of the reliability and generalizability of this information.

## Methods

Details of the literature search, inclusion criteria and methods in data extraction are reported in the companion paper (Mallett et al [7]). To identify articles, published subsequent to the original articles on model development, that might include external validations of the 47 prognostic models, we completed a citation search on each of the 47 original articles (30 December 2009). We used the Web of Science^® ^citation search (citation databases: Science Citation Index expanded, Social Sciences Citation Index, Arts & Humanities Citation Index and Conference Proceedings Citation Index - Science 1990 onwards) through the ISI Web of Knowledge^SM ^(Thomson Reuters 2009). Titles and abstracts for the cited references were screened and where appropriate full articles (SM).

### Validity assessment and data extraction

Topics covered included: number of patients and events, source of patients, endpoints for analysis, methods and reporting of multivariate analysis, numerical and graphical presentation of model, creation of prognostic index and risk groups, model discrimination and calibration, methods of validation and usability of reported model. In a companion paper (Mallett et al [7]) we report the assessment of methods and reporting, including study design, sample size, number of patients and events, outcome definition, number and coding of variables in model, methods of selection of variables.

Twenty items were extracted by duplicate data extraction by two of three reviewers (SM, SD, RW) with reference to a third reader where necessary. One reviewer (SM) assessed all articles and all items. For eight items examination of disagreements led to refinement of data items and re-assessment by a single reviewer (SM) due to study resource and timeline limitations. If more than one model was presented in the article, the first reported in the title, abstract or text was selected.

## Results

We assessed 47 articles in which prognostic models of cancer were developed for methods and reporting of the prognostic model performance [8-54]. A detailed description of the characteristics of these studies is reported in an accompanying paper (Mallett et al [7]).

Across 20 data extraction items relating to reporting of model performance measures, there was agreement in 76% of the items between readers. Over half of the differences were caused by ambiguities in the articles, the definition of the data item or where the disagreement required reference to a third reader to resolve. Examples of items frequently referred to a third reader included which methods used to create risk groups should be classified as *data driven*; and how much information from the final model was used to derive the prognostic index.

### Reporting of model

Cox models were used in 94% (44) of studies (Table 1) of which three articles included an additional modelling method (two on recursive partitioning analysis, one on artificial neural networks) [10,36,48]. Of the three articles where a Cox model was not used, a Weibull model was used in one article [39] and recursive partitioning analysis in another [25], and in a third article Cox modelling was rejected, but the method used was not reported [12]. The assumption of proportional hazards was reported as tested in 10 of the Cox models [8,12,21,22,29,33,35,40,45,49].

**Table 1:** Numerical and graphical presentation of model (n = 47)

	% (n) articles
Statistical model used	
Cox only	88 (41)
Cox plus other (two RPA, one ANN)	6 (3)
Other (one Weibull, one RPA, one unclear)	6 (3)
Assumption of proportional hazards tested†	21 (10)

Final prognostic model reported*	96 (45)
Regression coefficient reported**	72 (34)

Reproducibility of model development assessed††	11 (5)
Model with same variables, not same coefficients	9 (4)
Model generating both new variables and coefficients	4 (2)

† In three articles assumption of proportional hazards was not applicable as models used were RPA (recursive partitioning analysis) and ANN (artificial neural network).

* Two articles did not report the final model

** Not applicable in two articles using RPA or ANN model

†† One article used both methods to examine model reproducibility.

The final model used to develop the prognostic index, score or to make prognostic statements was reported in 96% (45) of the articles (Table 1). The model coefficients (hazard ratio or log hazard ratio) were reported in 72% (34) of the articles. In two articles numerous models were presented but not the model used to develop the prognostic index [50,52].

### Reproducibility of model development

Evaluation of model development methods in terms of both variable selection and coefficient estimates often reveals very different models can be selected based on bootstrap resampling of the patient dataset [55,56]. Where intermediate steps are used in model development, such as testing interaction terms or collapsing categories of variables, it might not be practicable to validate all model building steps fully [57].

In five articles the reproducibility of model development was examined (Table 1), in two articles researchers reselected the variables in the model [18,33], and in four articles the coefficient values were refitted [33,38,39,45]. Four of these articles also included validation of the final model in addition to evaluation of model reproducibility [18,33,39,45].

### Development of prognostic index

Where a prognostic model is based on a large sample size and relevant variables are included in the final model, reasonable estimates of the coefficient values for each variable are likely. The prognostic index is developed as a sum of the variables from the model, weighted by their coefficient values (log hazard ratio values). If the model was developed from a small sample, coefficient values in the model are likely to be unreliable, partly due to idiosyncrasies in data that the model is developed from rather than generalizable patterns [58]. Validation of prognostic models, either internally (using the same data) or externally (using different data), is essential to understand the reliability of both the choice of variables and the values of coefficients for each variable.

The development of a prognostic index was reported in 81% (38) of the articles (Table 2). Of the nine studies where a prognostic index was not developed, four studies included risk groups [19,25,36,48] and in five studies a model was developed but neither prognostic index nor risk groups were developed from the model [10,21,22,42,54].

**Table 2:** Prognostic index, risk groups and model fitting

	% (n) articles
Prognostic index (PI) developed	81 (38)
Components of final model used to create PI	
Same variables and coefficients	34 (13/38)
Same variables but not same coefficients	21 (8/38)
Neither same variables nor coefficients	29 (11/38)
Method unclear	16 (6/38)

Risk groups are created from prognostic model	76 (36)
Method used to create risk groups	
Data driven	28 (10/36)
Equal size groups created	14 (5/36)
Other non data driven method	11 (4/36)
Method unclear	8 (3/36)
Method not reported	39 (14/36)
Number of risk groups created	
Two risk groups	11 (4/36)
Three risk groups	39 (14/36)
Four risk groups	31 (11/36)
Five or more groups	11 (4/36)
Several different risk groupings used	8 (3/36)

In the 38 articles where a prognostic index was developed, the final model was not reported in two articles [50,52]. In nine articles the coefficients of the final model were not reported [13,18,20,29,32,39,44,50,52].

Appropriate methods for construction of a prognostic index from the final model were used in 34% (13 of 38) of articles [8,14-16,23,27,33,35,38,40,43,47,51], where the prognostic index was developed as the sum of the variables from the model, weighted by their coefficient values (log hazard ratio) (Table 2). In six articles the methods used to develop the prognostic index from the final model were not reported or were unclear [17,24,26,32,39,44].

In 21% (8 of 38) of the articles, the variables from the final model were used to develop the prognostic index but not the coefficient values [9,12,20,28,34,44,46,49], although in five of these articles authors stated their intention to use the coefficient weightings from the final model. In these eight articles that used the same variables as the final model, the following differences to the appropriate modelling methods were reported: counting factors, where equal weighting is applied to each variable was used in four articles [12,28,44,46]; a different weighting of a single variable from the weighting in the final model was assigned in one article [34]; coefficients from the univariable analysis instead of from the multivariable final model were used in one article [20]; a negative sign was missing from the coefficient in the prognostic index in one article [49]; score weightings that did not correspond to the order of the coefficients from the final model were assigned in one article [9].

In 11 articles researchers used neither the same variables nor coefficients as reported in the final model, for development of the prognostic index. In these articles researchers reported using the following differences to the appropriate methods: in two articles a previously published prognostic index was modified by addition of a new variable and weightings assigned to factors were derived from two different models [37,52]; between one and three variables were added into the prognostic index that were not included in the final model in five articles [11,30,45,52,53]; non-significant variables were present in the final model, but were not included in the prognostic index in three articles [17,24,41]; researchers described 'adjusting for 10 non significant variables' without including these variables in the final model in one article [13]; a significant variable was dropped from the final model from the prognostic index without explanation in one article [31]; in two articles researchers changed how variables are coded between the final model and prognostic index without apparently re-running the model to get new coefficients [13,31]; in four articles researchers derived the final model by counting variables, effectively assigning equal weighting to all factors regardless of coefficients in the model [11,13,17,30]. In four studies researchers used two of these methods together [17,30,31,52], and in one study three of these methods were used [13].

In 95% (36 of 38) of the studies developing a prognostic index, authors reported the number of variables used, corresponding to a median of four variables (IQR 3 to 5, range 2 to 9).

### Development of risk groups

There is no consensus on how to create risk groups, or how many risk groups to use [59]. Risk groups can be created directly from the model or by grouping prognostic index scores into risk groups. Even where there is fair consensus on which patients would be classified as having high risk or low risk, often for clinical purposes physicians are most interested in reclassification of patients at intermediate risk, for whom treatment decisions are unclear [60]. A disadvantage of classifying risk into only two groups as opposed to three or more risk groups, is that readers of the model are unable to see how risk changes across risk groups or to estimate risks for alternative risk groups from those chosen by the original modellers.

In the absence of an *a priori *clinical consensus on cutpoints for prognostic risk groups, then the currently preferred method is to use a non data driven method to assign risk groupings. These methods include splitting the population into equal size groups such as thirds or quarters. This is an equally arbitrary approach but more efficient in terms of sample size than splitting the prognostic index into equal intervals, which may result in a very small number of patients in extreme risk groups.

Data driven approaches are likely to considerably overestimate model performance and are not advised. Two data driven approaches are frequently used. The minimal *P*-value approach leads to bias as it uses multiple testing to find an optimal cut point in terms of study results for a given data set [61,62]. The post hoc alteration of risk group cutpoints based on study results, such as a combination of risk groups similar on Kaplan Meier plots, can lead to bias as hazard coefficients are not invariant across different cutpoints of an outcome variable [63]. Similarities can be seen in post hoc alterations to the cutpoint of a diagnostic test, and how this can bias diagnostic accuracy results [64].

Risk groups were developed from the prognostic model or prognostic index in 76% (36) of studies (Table 2). In nine studies researchers used non data driven methods to develop risk groups; five used equal sized groups [9,26,31,33,38]; two used cutpoints from previous publications [39,52]; one used arbitrary percentiles without justification [49]; and one used categories of prognostic index [44]. In 10 studies risk groups were created using data driven methods that are likely to overestimate the separation of prognostic groups when the model is validated on external datasets, in nine by combining prognostic index scores or recursive partitioning model termini with similar risk [15,17,23,25,27,30,35,36,48], in one by using a minimal *P*-value approach [47]. The methods used to develop risk groups were not reported or are unclear in 17 studies.

### Discrimination and calibration

The discrimination of a prognostic model indicates how well the model separates patients who experience an event of interest from those who do not [65,66]. Discrimination can be presented graphically by a Kaplan Meier (KM) plot of survival for patients in different risk groups.

Several measures of discrimination have been developed including the R squared [3], D statistic [67], c-index [4], SEP and PSEP [68,69], K [70], NRI [6], IDI [6] and decision curve analysis [71]. Some of these tests and measures can only be applied to comparisons between categorical groups such as risk groups (for example, log rank, NRI) whereas others can be applied to continuous measures such as prognostic indices (ISI, c-index, D). Some of the methods used to assess discrimination and calibration of a logistic regression model cannot be applied to Cox models and vice versa [72].

The log rank test, although easy to implement alongside a KM graph, does not give an estimate of the magnitude of the separation of the risk groups but is used to test for a difference in survival between risk groups. The use of *P-*values should be avoided as *P*-values are not useful measures of how well a model separates patients with and without events [69].

The discrimination ability of a prognostic model can be presented for the data used to develop the model (Table 3) although these measures are more important in understanding the performance of models in internal and external validation (Table 4).

**Table 3:** Model performance on data used to develop model and usability* (n = 42)

	Articles with risk groups (n = 36)	Articles with PI but no risk groups (n = 6**)
Presentation of discrimination of model predictions†		
KM for risk groups	34	NA
Nomogram	2	4
Other graphical	2	2
% survival probability at fixed time††	22	0
Index of discrimination (see below)	9	2
Log rank	17	NA
Unspecified *P*-value	6	0
No presentation	0	0

Index of discrimination$		
c-index	7	1
R squared or goodness of fit or Brier score	1	1
D	0	0
Other - K (Begg), sensitivity and specificity	2	0
Reclassification of patient risk	0	0

Calibration		
Yes	1	1
No	35	5

Model usability from article$$		
Prognostic score or risk group can be assigned	33	6
Survival presented for risk group and/or prognostic score	36	5
Instructions for use suitable for physicians included	3	3

* Five articles did not develop PI or use risk groups.

**Four of six articles had some commonality: three articles included the same author, one the same department.

†More than one option possible.

†† All articles have either KM or % survival by risk group.

$ Two articles with risk groups report two indices of discrimination.

$$ Four articles are unusable, lacking one or other criteria.

**Table 4:** Model performance on validation data

	Articles with risk groups (n = 16)
Presentation of discrimination of model predictions	
KM for risk groups	1
Other graphical	0
% survival probability at fixed time	2
Index of discrimination (see below)	11
Log rank	1
Unspecified *P*-value	0
No presentation of discrimination	4

Index of discrimination$	
c-index	10
R squared or goodness of fit	4
D	0
Other - k (Begg), SEP (Graf)	2
Reclassification of patient risk	0

Calibration	
Yes	2
No	14

† More than one option possible

$ Two studies reported two indices of discrimination, one study three indices

Table 3 summarises the presentation of discrimination for the original dataset used to develop the model in our sample of articles. Ninety-four percent (34 of 36) of studies that developed risk groups for a prognostic index presented differences in survival between risk groups using Kaplan Meier plots. The log rank test was reported in 17 studies. The percentage survival probability at a fixed time in the different risk groups was reported in 22 studies. In nine studies a measure of discrimination was reported, in seven studies the c-index was used [14,15,33,34,46,47,49] and in two studies other discrimination measures were presented [38,39] (Table 3).

Model calibration describes how well the estimates of survival from the model correspond to the survival from the observed data [66,73] and can be described as a measure of the extent of bias in a model [74]. Calibration in Cox models can be presented at a specific time point, as a plot of observed proportions of events against predicted probabilities in a new dataset often based on 10^ths ^of risk groups [75]. In logistic regression models the Hosmer-Lemeshow test can be used, but this as a single test does not give information on how individual risk groups (for example, each 10^th ^of risk group) is calibrated and it has limited statistical power to assess poor calibration and is over sensitive with very large samples. We accepted model calibration on the model development dataset as presented if the percentage survival in risk groups at a fixed time point was shown for both the model predictions and the observed data.

Researchers in only one study presented calibration of the model on the model development data, at a fixed time point, as a comparison of model predictions of percentage survival in risk groups with actual survival percentages [34]. It is unclear how censored data are treated in the actual survival prediction data.

### Usability of model

We also assessed how explicit and usable the model was for those wanting to apply the model. For a model to be usable by others, we required sufficient reporting to enable a reader to compute a score or risk group, and in addition information to link this to survival probability. Ninety percent (38 of 42) of the studies fulfilled both of these requirements for a usable model (Table 3). In this assessment, the predicted survival lines in nomograms were included as providing information from the model on survival according to the prognostic score. However, instructions likely to be suitable for physicians on how to use the prognostic model, either as specific instructions or as a worked example, were included in only six articles [8,9,18,32,39,45]. In two articles [18,32] example text for the physicians to explain to patients the interpretation of their scores was also included.

### Model validation

Evaluation or validation of a prognostic model is a process of establishing that a model works satisfactorily for patients other than the original dataset used to develop the model [69]. Model validation uses the same model (that is, the same variables and same coefficients or, equivalently, the original prognostic index) to evaluate both discrimination and calibration of model predictions with observed patient outcome in new data [66].

Internal validation refers to evaluation in the same patient data, although sometimes the term internal validation includes evaluation in different patients from the same patient population. For internal validations on the same patients, methods such as bootstrapping or jackknife methods are used. Where internal validation uses data within the same population, methods include split sample and cross validation [76]. In split sample validation the data is split into a model development and testing dataset. Cross validation is an extension of split sampling methods, but where the sample split is repeated so all patients have served once in the model evaluation dataset. Although split sample and cross validation methods use different patient data to that used to develop the model, the new data is often closely related or a random split of the same dataset. Split sample methods and cross validation with fewer than 10 repeats, have been reported to provide an inferior validation method to bootstrapping for many reasons, including inefficient use of data leading to less stable model development, poor performance and bias [77]. The most stringent form of validation is external validation, where the generalizability or transportability of the model is evaluated in new patients in a separately collected population.

Model validation was reported in addition to model development in 34% (16) of studies [8-10,16,18,25,32,33,39,40,45-47,49,51,54]. In 15 studies, researchers validated using data from the same population; six used the bootstrap method [33,39,45,46,49,51], five used a random split [8-10,16,47], five cross validation [10,18,25,39,40], two temporal split (Table 5). Just 11% (5) of articles included external validation with data from a different population setting [18,32,33,39,46].

**Table 5:** Reproducibility and validation of models

Topic	% (n = 47) articles
Model validation included	34 (16)
Validation dataset*	
Same data (bootstrap)	13 (6)
Same population, new data**	23 (11)
External (that is, new population setting)	11 (5)
Larger series including original sample	0 (0)

Validation of models	
Final model with same coefficients and variables	26 (12)
Unclear reporting	9 (4)

Modifications suggested to model in light of validation?	0 (0)

* Five studies included validation with more than one dataset.

** Methods used by studies were as follows: five studies used a random split, two used temporal split, five used cross validation, one used the jacknife method. Two studies used two and three methods respectively.

We assessed the types of dataset used in the five articles (Table 5) where external validation was reported as part of the original model development article: RCT datasets were used in two studies [33,39], a retrospective database was used in three studies [18,32,46], an external validation set with some patients from an RCT and some from a consecutive patient series were used in one study [33]. In two studies researchers used external validations from the same hospital; one with RCT data [33], one with different treatments [46]. Three studies used external validation on patient data from a different hospital [18,32,39].

In 13 articles the number of patients in the validation datasets were reported (median 200, IQR 148 to 359, range 5 to 1,782). In nine articles the number of events in validation datasets were reported (median 110, IQR 65 to 149, range 15 to 574) [16,18,32,33,39,45,46,49,51].

Discrimination of the model in the validation dataset was presented in 75% (12 of 16) of the articles including validation (Table 4) [8,9,16,18,25,33,39,45-47,51]. In 11 studies one or more indices of discrimination were reported, with the c-index reported in 10 studies and *goodness of fit P*-values (AIC, BIC, Cox model fit) in four studies.

In only four percent (2) of models had researchers presented any information on model calibration (Table 4) [9,18]. In these studies calibration plots were reported at a fixed time point for model predictions of percentage survival against actual survival percentage. None of these 16 model validations resulted in any recommendations to modify the prognostic model in the light of the validation. General rules for the need to update prognostic models before clinical application have not yet been established [73,78].

In addition to the five articles in our review that included external validations, we also searched for subsequent publications that included external validations for the 47 prognostic models, using a citation search in December 2009. For eight prognostic models [18,20,29,34-36,46,47] subsequent articles have been published that used external patient data and reported completion of a model validation [79-92]. For three models, a model evaluation was reported in one subsequent article per model [29,35,36] whereas in five models, evaluation was reported by more than one article [18,20,34,46,47]. The same authors as had developed the prognostic model had published reports of evaluation for two models [29,36], whereas different authors reported evaluation for six models [18,20,34,35,46,47]. Overall, in the same or subsequent publications, 21% (10 of the 47) of models were reported as evaluated using external datasets, although the quality of evaluations was often poor and uninformative.

### Example of good methods and reporting

Although the quality of the articles was generally disappointing, we particularly wish to highlight one article using good methods and good reporting [33]. This study deserves mention as researchers included: reporting of the multivariable model and its coefficients, correct use of the multivariable to develop the prognostic index, creation of risk groups using preferred methods, (for example this study used equal size groups), presentation of the model in a form usable by others, both internal and external validation of the model. Kaplan-Meier plots are reported for the validation data by risk groups and a recognised discrimination measure is reported. This same article was also the best example for our companion article on developing prognostic models (Mallett et al [7]) making it a good example for those wishing to develop prognostic models to use in combination with books and articles providing good advice on methods in prognostic modelling [3-5,69,74]

## Discussion

This research has highlighted current practice in methods used to develop prognostic models for clinical predictions about the patients, and the measures of model performance reported. The quality of prognostic models depends on researchers understanding the assumptions inherent in the methods and following sound principles to ensure methods are appropriately applied [4]. Explicit reporting of methods and performance measures of models to other researchers is important to enable further model validation and transparent evaluation of clinical usefulness of models [93].

Very few articles in our study reported on how well model predictions performed, either in terms of discrimination, the ability of the model to separate patients with different outcomes, or by calibration, how accurately the model estimated the probability of outcome. Most statistical models are derived from a sequence of data driven steps leading to likely bias both in model development and the performance of a prognostic index or risk groups generated from it. Although there is no consensus on the best methods in several areas of prediction modelling, such as creation of prognostic groups, there is consensus that some methods are not advisable [61,94]. Unfortunately this study shows that these ill advised and biased methods are in widespread use, which will reduce the reliability of models and predictions of many prognostic models.

Though this research relates to prognostic models in cancer, problems identified in these prognostic models are not specific to cancer. Similar problems have been found in reviews of other areas of medicine [95]. This study included mostly Cox models, however the principles for reporting of logistic regression models are similar, even if some measures are different. Frequent use of poor methods have been reported in the development of logistic regression models [96]. A further limitation of our study was that only 47 articles were reviewed, however we judged little further value would be obtained from review of a larger number of articles.

### Reporting methods to develop risk groups

There is little guidance or consensus on how to develop risk groups from a prognostic index, however using groups with equal numbers of patients or based on a justifiable clinical reason is the preferred method. Using data driven methods (based on outcomes of the data analysis itself) is not advisable although we found these methods are frequently used (in 28% of models). Previous research has found similar inappropriate data driven methods were frequently used [59]. Preferred methods are those based on clinical consensus or arbitrary cutpoints such as quartiles of population.

In our research we found frequent use of highly biased methods to develop models and to derive prognostic indices and groups for prediction of patient risk. There are no specific guidelines on how to develop, measure performance and validate prognostic models, but there are some excellent books and articles providing advice on good and poor methodology [3-5,74].

### Lack of reporting of model performance

Reporting of model performance using discrimination and calibration measures was poor (Tables 3 and 4). Only two articles reported calibration data in external validation data (Table 4).

Previous research has also found that there is poor reporting of model performance in terms of discrimination and calibration measures in logistic regression models [96-98]. In prediction models in reproductive medicine with external validation, most models reported either discrimination or calibration [95]. Discrimination is frequently reported using the c-index, equivalent to the area under the ROC curve. The c-index measures the probability that two patients, one with an event and one without, will be ranked correctly. This c-index is not related to any particular prognostic index threshold, but is integrated across all possible thresholds, whether clinically applicable or clinically absurd. The clinical applicability and meaning of the c-index has been questioned recently [6]. Model *goodness of fit *tests are often presented with the model development and validation, but these tests do not indicate how well a model predicts patient outcome [72]. The newer methods that describe model discrimination in terms of patient reclassification between risk groups, are starting to be used in published studies and should provide more clinically relevant information to assess model performance [99].

### Internal and external validation

Validation of models is essential to establish whether a prognostic model is likely to provide useful classification of patient risk. External validation is an essential pre-requisite before models are applied in clinical practice, preferably by external investigators [66,76]. We found 34% of articles included some validation, but external data from a new patient population was used in only 11% of studies. Reported external evaluation of eight models was found in subsequent publications. In total only 21% (10 models) were reported as externally validated in either the original articles or in the subsequent four years. Other research has found that a large range, 0% to 52%, of articles where a prediction model was developed, included either internal or external validation [93,95-98].

Articles in this study did not report using multiple imputation methods to address missing prognostic variable data. Several articles confirm that only complete case data are included in model development, indicating the presence of selection bias in the model. Ongoing methodological research provides guidelines on the use of multiple imputation for missing data [100], development and validation of models with missing data [101] and how to apply models when missing data are present [102].

### The implications for clinical medicine

Prognostic models are developed to provide objective probability estimates to complement clinical intuition of the physician and guidelines [73]. Many published prognostic models have been developed using poor methodological choices that may adversely affect model performance. This may help to explain why so few models are used in clinical practice. Appropriate choice and use of prognostic models in clinical practice requires model validation and reporting appropriate measures of model performance in order to assess reliability and generalizability of models.

## Conclusions

Development, validation and assessment of the performance of prognostic models are complex, and depend on researchers understanding statistical methods and how to apply them appropriately. We found poor reporting of the methods used to develop models and details of the models. Questionable methods are widely used to develop prognostic indices and few models are validated, even using internal validation methods that do not require additional datasets.

## Abbreviations

AIC: Akaike information criterion; ANN: artificial neural network; BIC: Bayesian information criterion; DFS: disease free survival; EPV: events per variable; IQR: interquartile range; KM: Kaplan Meier plot; MeSH: Medical subject heading from U.S. National Library of Medicine's controlled vocabulary; NLM: National Library of Medicine; PI: prognostic index; RCT: randomised controlled trial; RPA: recursive partitioning analysis.

## Competing interests

The authors declare that they have no competing interests.

## Authors' contributions

SM contributed to design, carried out data extraction on all articles and items, compiled results and drafted the manuscript. PR and DGA contributed to the design and drafting of the article. RW and SD carried out duplicate data extraction for some data items and commented on the manuscript.

## Authors' informations

All authors are medical statisticians.

## Pre-publication history

The pre-publication history for this paper can be accessed here:

http://www.biomedcentral.com/1741-7015/8/21/prepub
